# Primary tracheal small‐cell carcinoma detected 11 months after surgery for pulmonary large‐cell neuroendocrine carcinoma: A case report

**DOI:** 10.1111/1759-7714.14860

**Published:** 2023-03-28

**Authors:** Chihiro Sugimoto, Shuhei Teranishi, Tomoe Sawazumi, Satoshi Nagaoka, Hirokazu Nagayama, Wataru Segawa, Shuntaro Hiro, Yukihito Kajita, Chihiro Maeda, Sousuke Kubo, Kenichi Seki, Ken Tashiro, Nobuaki Kobayashi, Masaki Yamamoto, Makoto Kudo, Takeshi Kaneko

**Affiliations:** ^1^ Respiratory Disease Center Yokohama City University Medical Center 4‐57 Urafune‐cho, Minami‐ku Yokohama 232‐0024 Japan; ^2^ Division of Pathology Yokohama City University Medical Center 4‐57 Urafune‐cho, Minami‐ku Yokohama 232‐0024 Japan; ^3^ Department of Pulmonology Yokohama City University Graduate School of Medicine 3‐9 Fukuura, Kanazawa‐ku Yokohama 236‐0004 Japan

**Keywords:** accelerated hyperfractionated radiotherapy, chemotherapy, nasal high‐flow therapy, primary tracheal small‐cell carcinoma

## Abstract

Primary tracheal small‐cell carcinoma is rare, and is often treated using small‐cell lung cancer guidelines given that no standard treatment has been established for it. We report a patient in whom nodules appeared in the trachea and left main bronchus 11 months after surgery for pulmonary large‐cell neuroendocrine carcinoma; a biopsy revealed small‐cell carcinoma. Given the absence of malignant lesions elsewhere in the body, the lesions were diagnosed as primary tracheal small‐cell carcinoma. Respiratory failure progressed rapidly owing to airway stenosis caused by the growing lesion, and the patient required nasal high‐flow therapy. However, the lesions shrank a few days after commencing first‐line chemotherapy, and his respiratory failure resolved. Accelerated hyperfractionated radiotherapy was administered in conjunction with the third course of chemotherapy, and the patient ultimately achieved a complete response. Although the lesions were initially suspected of being postoperative recurrence of pulmonary large‐cell neuroendocrine carcinoma, the fact that the biopsy revealed them to be primary tracheal small‐cell carcinoma indicates that intra‐airway nodules that appear after lung cancer surgery may possibly be primary tracheal tumors.

## Graphical abstract

We report a patient in whom nodules appeared in the trachea and left main bronchus 11 months after surgery for pulmonary large‐cell neuroendocrine carcinoma; a biopsy revealed small‐cell carcinoma. Four courses of chemotherapy, plus accelerated hyperfractionated radiotherapy with the third course, resulted in a complete response. Physicians must consider that intra‐airway nodules that appear post lung cancer surgery may be primary tracheal tumors.

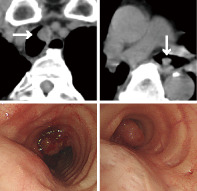

## INTRODUCTION

Primary tracheal tumors are rare; among them, primary tracheal small‐cell carcinomas (SCCs) are very infrequent, and there are no large‐scale studies exploring their treatment.^1^, ^2^, ^3^ Individual case reports suggest that surgical resection, chemotherapy, radiotherapy, and chemoradiotherapy protocols used for patients with lung SCC can also be administered to patients with primary tracheal SCC; however, the optimal treatment strategy remains unknowns. We report a patient in whom primary tracheal SCC was detected 11 months after surgery for pulmonary large‐cell neuroendocrine carcinoma (LCNEC), and in whom complete response was achieved following chemotherapy plus accelerated hyperfractionated radiotherapy (AHF).

## CASE REPORT

**FIGURE 1:**
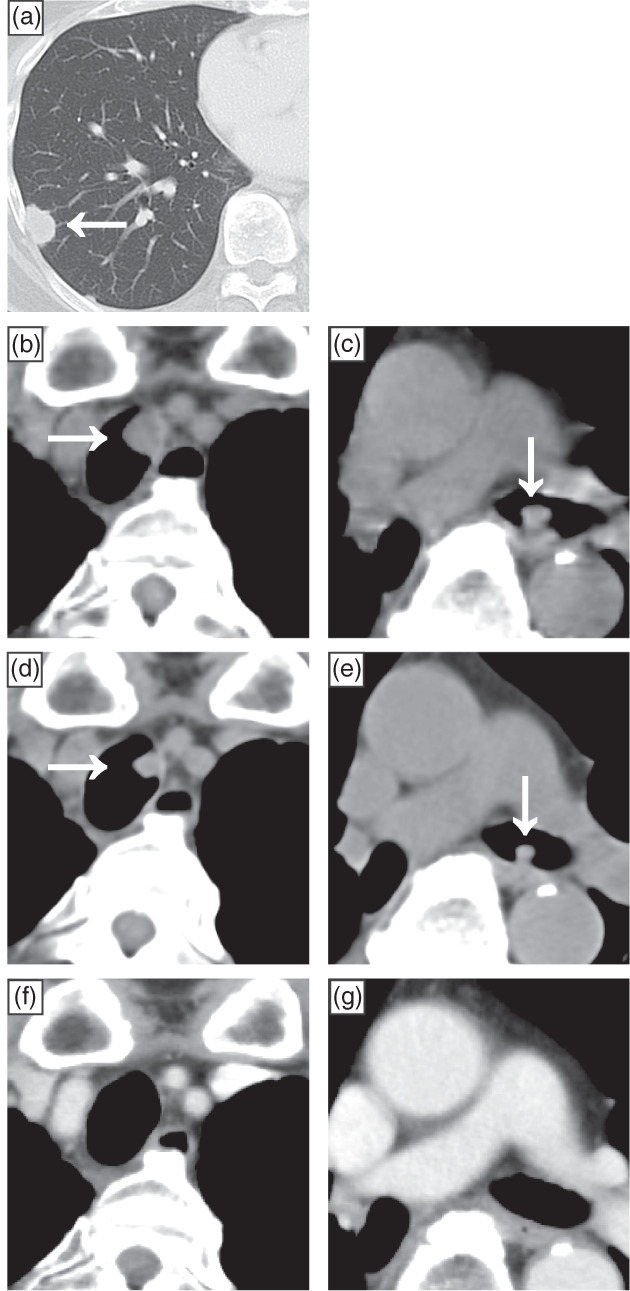
Before surgery, computed tomography (CT) showed a nodule in the lower lobe of the right lung (a). Eleven months after thoracoscopic lower lobectomy of the right lung, CT showed nodules in the trachea (b) and left main bronchus (c). CT performed 6 days after the first course of first‐line chemotherapy revealed that these nodules had shrunk (d, e). CT after four courses of first‐line chemotherapy (with the addition of accelerated hyperfractionated radiotherapy starting with the third course) showed that the nodules in the trachea and left main bronchus had disappeared (f, g).

**FIGURE 2:**
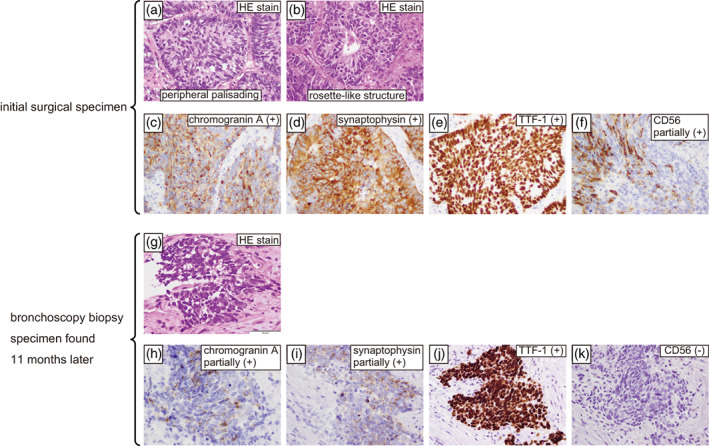
Microscopic (hematoxylin–eosin staining) and immunohistochemical analysis of the initial surgical specimen (a–f) and of the bronchoscopy biopsy specimen found 11 months later (g–k). Hematoxylin and eosin staining showed that tumor cells in the surgical specimen had rich cytoplasm with prominent nucleoli as well as peripheral palisading and a rosette‐like structure (a, b). On immunohistochemistry, the surgical specimen was positive for chromogranin A (c), synaptophysin (d), and thyroid transcription factor‐1 (e), and was partially positive for CD56 (f). Hematoxylin and eosin staining showed that tumor cells in the bronchoscopy biopsy specimen were relatively small with a high nuclear/cytoplasm ratio, no prominent nucleoli, and scant cytoplasm (g). Immunohistochemistry was partially positive for chromogranin A (h) and synaptophysin (i), positive for thyroid transcription factor‐1 (j), and negative for CD56 (k). Magnification = 400× for all panels. HE, hematoxylin and eosin; TTF‐1, thyroid transcription factor‐1

A 77‐year‐old man with a 30‐pack‐year smoking history visited our hospital after an abnormal chest shadow was detected during a physical examination. Computed tomography (CT) showed a nodule in the lower lobe of the right lung (Figure 1a). He underwent thoracoscopic lower lobectomy of the right lung. Pathological examination revealed a malignant epithelial tumor composed of cells with prominent nucleoli and rich cytoplasm; peripheral palisading and rosette‐like structures were also observed (Figure 2a, b). On immunohistochemistry, the tumor was positive for chromogranin A, synaptophysin, and thyroid transcription factor‐1 and was partially positive for CD56 (Figure 2c–f), leading to a diagnosis of LCNEC pT1bN0M0 stage IA. At the patient's request, no adjuvant chemotherapy was administered.

**FIGURE 3:**
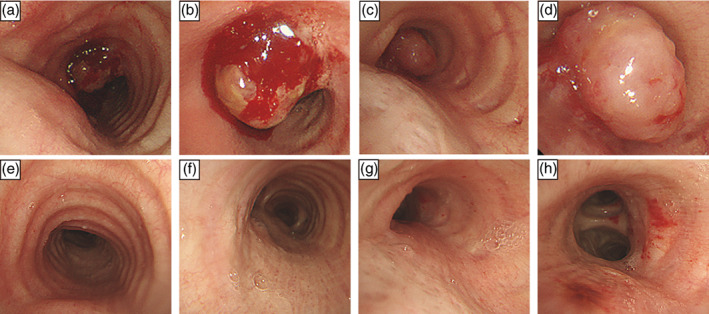
Bronchoscopy before commencing treatment showed nodules in the trachea (a, b) and left main bronchus (c, d). Bronchoscopy after four courses of first‐line chemotherapy (with accelerated hyperfractionated radiotherapy added starting with the third course) showed that the nodules in the trachea (e, f) and left main bronchus (g, h) had disappeared.

Eleven months after surgery, the patient developed bloody sputum and dyspnea on exertion, and CT revealed an 11‐mm intratracheal nodule (Figure 1b) as well as a 9‐mm left main bronchus nodule (Figure 1c). Bronchoscopy revealed an elevated lesion in the same area (Figure 3a–d), and a bronchoscopy biopsy was performed from the left main bronchial node. Pathological examination revealed a malignant epithelial tumor composed of relatively small cells with a high nuclear/cytoplasm ratio, inconspicuous nucleoli, and scant cytoplasm; no peripheral palisading or rosette‐like structures were identified (Figure 2g). Immunostaining showed partial positivity for chromogranin A and synaptophysin, positivity for thyroid transcription factor‐1, and negativity for CD56 (Figure 2h–k), leading to a diagnosis of SCC. After a systemic search revealed no malignant lesions in other parts of the body, the patient was diagnosed with primary tracheal SCC.

**FIGURE 4:**
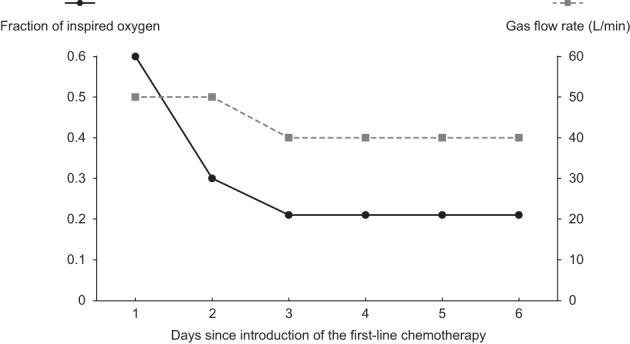
Chart showing the gas flow rate and fraction of inspired oxygen for nasal high‐flow therapy. On initiation of first‐line chemotherapy, the patient required a gas flow rate of 50 L/min and fraction of inspired oxygen of 0.6. Subsequently, the gas flow rate and fraction of inspired oxygen requirement rapidly decreased. After 6 days, the patient was weaned from nasal high‐flow therapy and no longer required oxygen.

Two days after bronchoscopy, the patient experienced rapidly progressing respiratory failure from airway stenosis caused by the growing lesion. Oxygenation could not be maintained despite use of a reservoir mask at a flow rate of 15 L/min, nasal high‐flow therapy (NHF) with a gas flow rate of 50 L/min and a fraction of inspired oxygen of 0.6 was introduced. First‐line chemotherapy with carboplatin (area under the curve = 5 mg/mL/min) and etoposide (80 mg/m^2^ body surface area) were also initiated on the same day. Six days after the start of chemotherapy, CT revealed shrinkage of the lesion (Figure 1d, e), and the patient was weaned off NHF and no longer required oxygen administration (Figure 4). The chemotherapy was changed to cisplatin and etoposide (both at 60 mg/m^2^ body surface area) for the second and subsequent courses, with AHF (45 Gray/30 fractions) introduced during the third course. After the fourth course of chemotherapy, the lesions had resolved on CT (Figures 1f, g) and bronchoscopy (Figures 3e–h). After achieving a complete response, prophylactic whole‐brain irradiation (25 Gray/10 fractions) was performed. Five months have passed since the completion of chemotherapy, and the patient remains alive with no evidence of recurrence.

## DISCUSSION

Primary tracheal tumors account for approximately 0.2% of all respiratory tumors,^1^, ^2^ and include adenoid cystic carcinoma, squamous cell carcinoma, and SCC (comprising 34%, 31%, and 2.0% of such tumors, respectively).^3^

Treatments administered to the few reported patients with primary tracheal SCC included surgical resection, radiotherapy, chemotherapy, and chemoradiotherapy (Table 1).^4^, ^5^, ^6^, ^7^, ^8^, ^9^, ^10^ Our patient's lesion was confined to the airway; per the guidelines for limited‐stage lung SCC, the first treatment choice is AHF combined with early concurrent chemotherapy.^11^ The patient's requiring of NHF rendered irradiation difficult at the start of treatment. Since the response rate to first‐line chemotherapy for extensive‐stage lung SCC is as high as 78%–84%,^12^, ^13^ it was determined that chemotherapy alone would likely shrink the lesion and improve airway stenosis. A few days after the start of chemotherapy, the tumor had shrunk and respiratory failure had abated; a complete response was achieved after administering AHF during the third course.

**TABLE 1:** Details of the seven reported patients with primary tracheal small‐cell carcinoma

Case	Age	Sex	Chief complaint	LS/ES	Treatment	Response	Reference
1	67	Male	Cough, dyspnea	LS	Concurrent chemoradiotherapy, tracheal stent	UN	4
2	27	Male	UN	LS	Chemotherapy + radiation (whether concurrent or sequential is UN)	CR	5
3	41	Male	Dyspnea, wheezing	ES	Bronchoscopic electro‐surgery, concurrent chemoradiotherapy	CR	6
4	70	Female	Cough, dyspnea	LS	Chemotherapy followed by radiation	CR	7
5	35	Female	Dyspnea	LS	Concurrent chemoradiotherapy	CR	8
6	UN	UN	UN	UN	Chemotherapy + radiation (whether concurrent or sequential is UN)	CR	9
7	58	Female	Dyspnea, wheezing	LS	Resection, chemotherapy followed by radiation	CR	10

Abbreviations: CR, complete response; ES, extensive‐stage; LS, limited‐stage; UN, unknown.

Additionally, ablation, snare, cryotherapy, laser photo resection, electro‐surgery, argon plasma coagulation, and endotracheal stenting have reportedly been performed to treat the symptoms of airway narrowing associated with primary tracheal tumors.^4^, ^5^, ^6^, ^14^, ^15^, ^16^ However, these techniques require advanced preparation and cannot be performed immediately. It was difficult for our patient to undergo these procedures because respiratory failure rapidly worsened. We maintained oxygenation via NHF until the lesion shrunk with chemotherapy. This suggests that NHF may effectively manage patients experiencing respiratory failure because of airway stenosis caused by primary tracheal SCC until the patient responds to anticancer treatment.

Taken together, our patient's course suggests that physicians must carefully consider the possibility that intra‐airway nodules that appear post‐lung cancer surgery may be primary tracheal tumors.

## AUTHOR CONTRIBUTIONS

S.T. and C.S: writing – original draft. T.S., S.N., H.N., W.S., S.H., Y.K., C.M., S.K., K.S., and K.T: writing – review and editing. N.K., M.Y., M.K., and T.K: visualization and supervision. All authors approved the final version of the manuscript to be published.

## CONFLICT OF INTEREST STATEMENT

The authors have no conflicts of interest to declare.
